# Efficacy of Repeated Administration of Cultured Human CD34^+^ Cells Against Streptozotocin-Induced Diabetic Nephropathy in Rats

**DOI:** 10.3390/cells14221766

**Published:** 2025-11-11

**Authors:** Takayasu Ohtake, Amankeldi A. Salybekov, Tsutomu Sato, Shigeaki Okamura, Masaki Yazawa, Yuki Yano, Mehdi Hassanpour, Mitsuru Yanai, Makoto Imagawa, Takayuki Asahara, Shuzo Kobayashi

**Affiliations:** 1 Regenerative Medicine, The Center for Cell Therapy & Regenerative Medicine, Shonan Kamakura General Hospital, 1370-1 Okamoto, Kamakura 247-8533, Japan; 2 Kidney Disease and Transplant Center, Shonan Kamakura General Hospital, 1370-1 Okamoto, Kamakura 247-8533, Japan; shuzo@shonankamakura.or.jp; 3 Regenerative Medicine, Shonan Research Institute of Innovative Medicine (sRIIM), Shonan Kamakura General Hospital, 1370-1 Okamoto, Kamakura 247-8533, Japan; amansaab0@gmail.com (A.A.S.); t_satou@shonankamakura.or.jp (T.S.); okamura-shigeaki@nipro.co.jp (S.O.); yazawa.tokai@gmail.com (M.Y.); y_yano2@shonankamakura.or.jp (Y.Y.); m_hassanpour@shonankamakura.or.jp (M.H.); t_asahara@shonankamakura.or.jp (T.A.); 4 Department of Pathology, Sapporo Tokushukai Hospital, 1-1-1 Oyaji Higashi, Sapporo 004-0041, Japan; mmayamm@gmail.com; 5 Department of Pathology, KKR Sapporo Medical Center, 3-40 Ichijyo Rokuchome, Sapporo 062-0931, Japan; imagawa.kkr@gmail.com; 6 Cell Processing and Cell/Genome Analysis Center, The Center for Cell Therapy & Regenerative Medicine, Shonan Kamakura General Hospital, 1370-1 Okamoto, Kamakura 247-8533, Japan

**Keywords:** CD34^+^ cell, diabetic nephropathy, streptozotocin, kidney transcriptomics

## Abstract

**Highlights:**

**What are the main findings?**

1. Human cultured CD34^+^ cells improved urinary protein excretion and pathological damage in rats with diabetic nephropathy.
2. Human cultured CD34^+^ cells upregulated the expression of anti-inflammatory and angiogenesis-related genes in rat kidney tissues.

**What are the implications of the main findings?**

1. Human CD34^+^ cells have a regenerative effect and the potential to improve diabetic nephropathy.
2. The results of this study support the validity of future clinical trials using CD34^+^ cells to treat diabetic nephropathy.

**Abstract:**

To date, no clinical trial has investigated the potential of CD34^+^ cells to treat diabetic nephropathy. This study examined the efficacy of human CD34^+^ cells against diabetic nephropathy in rats. Rats were administered streptozotocin (STZ) intraperitoneally and divided into three groups: normal control, STZ control, and STZ plus cell therapy. The STZ-plus-cell-therapy group was administered human umbilical cord blood-derived CD34^+^ cells weekly for three weeks. At eight weeks, the rats’ renal function, pathology, and transcriptome profiles were assessed. Although blood glucose levels did not differ between the STZ-administered groups, urinary albumin excretion was significantly lower at 6 weeks in the STZ-plus-cell-therapy group than in the STZ control group (*p* < 0.001). Serum creatinine levels tended to be higher in the STZ control group and lower in the STZ-plus-cell-therapy group. Cell therapy significantly improved mesangial expansion, interstitial fibrosis, peritubular capillary rarefaction, and glomerular macrophage infiltration compared with the STZ control (*p* < 0.0001). Kidney transcriptomics revealed significant upregulation of genes related to M2 macrophage markers, cell homing, and angiogenesis in the STZ-plus-cell-therapy group. In rats with STZ-induced diabetic nephropathy, human CD34^+^ cells ameliorated renal injury through their anti-inflammatory and pro-angiogenic effects.

## 1. Introduction

Chronic kidney disease (CKD) is a progressive disease affecting more than 750 million individuals worldwide, presenting a major global public health burden [1]. CKD markedly increases the risk of mortality due to comorbid cardiovascular diseases and advanced renal failure. Among the various causes underlying CKD, diabetic nephropathy is a leading contributor to end-stage renal disease. Each year in Japan, nearly 40% of the 40 million incident hemodialysis patients start renal replacement therapy due to diabetic nephropathy [2]. However, therapeutic strategies to delay CKD progression remain limited [3,4,5,6].

Cell therapies have been employed in patients with intractable diseases, including chronic ischemic heart disease [7], chronic limb-threatening ischemia [8], and cerebrovascular ischemia [9]. CKD is a similarly important therapeutic target for regenerative therapy. Most regenerative therapies utilize mesenchymal stromal cells (MSCs) due to their immunomodulatory, anti-inflammatory, and vasculogenic potential [10]. In this context, CD34^+^ hematopoietic stem cells and endothelial progenitor cells (EPCs) may be powerful therapeutic alternatives thanks to their strong angiogenic and anti-inflammatory effects [11].

Most cell therapy studies for diabetic nephropathy, both preclinical and clinical, have employed MSCs [12]. Although a few preclinical studies have reported the efficacy of CD34^+^ cells for diabetic nephropathy [13,14,15], clinical trials are currently lacking. We recently demonstrated the efficacy of CD34^+^ cells in nondiabetic chronic progressive tubulointerstitial kidney injury induced by adenine feeding [11]. Repeated administration of CD34^+^ cells substantially ameliorated kidney injury. The administration of CD34^+^ cells markedly improved tubulointerstitial injury, interstitial fibrosis, and macrophage infiltration. Notably, peritubular capillary (PTC) density was well preserved in the CD34^+^ cell therapy group compared with the adenine control group. Both the angiogenic and anti-inflammatory potential of CD34^+^ cells are critical for inhibiting the progression of nondiabetic CKD. Building on the beneficial effects of CD34^+^ cells in an animal model of nondiabetic CKD, we conducted a translational clinical trial of autologous CD34^+^ cell therapy in patients with chronic progressive nondiabetic kidney disease. Although the results were preliminary, the CD34^+^ cell therapy improved the slope of the estimated glomerular filtration rate (eGFR) from negative to positive in three out of four patients with a progressive decline in the eGFR [16].

Accordingly, we hypothesized that the robust angiogenic and anti-inflammatory potential of CD34^+^ cells could be effective in treating diabetic nephropathy. Given the clinical need for effective therapies, we examined the potential of CD34^+^ cell therapy in an animal model of diabetic nephropathy. In consideration of the chronic and progressive nature of diabetic nephropathy and the purpose of future clinical application, we selected repetitive administration of human CD34^+^ cells, not a single injection, for diabetic nephropathy. Confirming the efficacy and mechanisms of CD34^+^ cells in animal models could pave the way for translational trials in patients with diabetic nephropathy.

## 2. Materials and Methods

### 2.1. Cell Preparation

With the support of gynecologists at our hospital, human umbilical cord blood (UCB)-CD34^+^ cells were obtained upon delivery from pregnant women who provided informed consent (approval number: SKEC-21-24). UCB-CD34^+^ cells were collected from the umbilical cord using a MiniMACS starting Kit and CD34 MicroBead Kit UltraPure human (Miltenyi Biotec, Leiden, Germany), plated at a density of 1 × 10^4^ cells/well in a 24-well tissue culture plate (BD Falcon, Bedford, MA, USA), and cultured for 7 days as described previously [11]. The cells were cultured in serum-free StemSpan SFEM (Stem Cell Technologies, Vancouver, BC, Canada) with an optimized combination of growth factors and cytokines (20 ng/mL thyroid peroxidase, 20 ng/mL interleukin-6, 100 ng/mL SCF, 100 ng/mL Flt-3 Ligand, and 50 ng/mL vascular endothelial growth factor). All reagents were purchased from PeproTech, Inc. (Rocky Hill, NJ, USA). This culture method (quality and quantity culture: QQc) was established previously and used to enhance vasculogenesis and proliferation and increase the colony-forming units of CD34^+^ EPCs [11]. The cell populations of pre- and post-QQc human UCB-CD34^+^ EPCs were evaluated by FACS (FACSVerseTM, BD, Franklin Lakes, NJ, USA). FlowJoTM (version 10.6, BD, USA) was used for data analysis.

### 2.2. In Vivo Experiment

**Figure 1:**
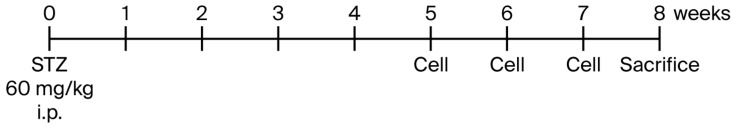
Study design. Streptozotocin (STZ; 60 mg/kg body weight) was intraperitoneally administered on day 0, and human umbilical cord blood-derived cultured CD34^+^ cells (1 × 10^6^/rat) were administered intravenously at weeks 5, 6, and 7 post-STZ administration in the STZ-plus-CD34^+^-cell-therapy group.

The study design is illustrated in Figure 1. Diabetes was induced in F344/N rats via intraperitoneal streptozotocin (STZ; 60 mg/kg). Rats with blood glucose levels > 300 mg/dL three days post STZ injection were fed standard laboratory chow and water and maintained under a 12:12 light/dark cycle. Body weight and blood glucose levels were monitored weekly, and 24 h urine samples were collected in metabolic cages at 0, 2, 4, 6, and 8 weeks post-STZ injection to calculate the urinary albumin/creatinine ratio (UACR). Eight weeks post-STZ administration, the rats were sacrificed, and blood samples were collected to determine creatinine levels. Pericardial cold saline perfusion was performed, and right kidney cortical tissue was harvested in RNlater for transcriptomic analysis. After right kidney sample collection, pericardial systemic perfusion using 4% paraformaldehyde was performed to obtain left kidney tissues for pathological and immunohistochemical evaluation. A total of 36 rats were used in the experiment and allocated to a normal control group (n = 10), an STZ-plus-vehicle (saline) control group (n = 14), or an STZ-plus-CD34^+^-cell-therapy group (n = 12).

### 2.3. Kidney Function and Pathological Evaluation

Serum creatinine (sCr) levels were measured at eight weeks by quantitative colorimetric creatinine determination using the QuantiChrom^TM^ Creatinine Assay Kit (DICT-500; Funakoshi Co., Ltd., Tokyo, Japan), as described previously [17]. Urinary albumin and creatinine levels were measured at Kamakura Techno-Science Inc., Kamakura, Japan. Urine creatinine levels were measured using the Modified Jaffe’s method and an Oxford Bio Medical Research Microplate Assay for Creatinine CR01, as described previously [18]. The urine albumin level was measured by ELISA using an LBIS Rat Albumin ELISA Kit AKRAL-120 (FUJIFILM Wako Pure Chemicals, Osaka, Japan) [19]. Kidney function was evaluated by serum creatinine levels and creatinine clearance at 8 weeks after STZ administration.

Harvested kidney tissues were fixed in 4% paraformaldehyde, transferred to 70% ethanol, embedded in paraffin, and sectioned into 2 μm thick sections. The sections were stained with periodic acid–Schiff and Masson’s trichrome (MT) stain to assess mesangial matrix expansion and interstitial fibrosis, respectively. The extent of mesangial matrix expansion was graded from 0 (minimum) to 4 (severe) in 50 glomeruli per rat, evaluating five randomly selected rats in each group. Interstitial fibrosis in the MT-stained sections was quantified as the fibrotic area per total area (%) and measured using an automatic image analyzer (CellSens, Olympus, version 1.11, Tokyo, Japan). Photographs of 20 fields under 200× magnification were obtained for each rat, with five rats per group.

### 2.4. CD31 and F4/80 Immunohistochemistry

As the CD31 antigen is constitutively expressed on the endothelial cell surface, the loss of CD31-positive staining most likely reflects capillary loss. We performed CD31 immunostaining to evaluate PTC loss using 3 μm thick paraffin sections. PTCs were identified by immunostaining with rabbit monoclonal anti-CD31 (PECAM-1) antibody (1:200; #77699; Cell Signaling Technology, Danvers, MA, USA). Immunostaining was performed using the BOND-III autostainer (Leica Biosystems, Wetzlar, Germany), according to the manufacturer’s protocol. Briefly, after deparaffinization of the paraffin sections, heat-induced epitope retrieval was performed using BOND Epitope Retrieval Solution 2 (prediluted, pH 9.0; Leica Biosystems, Wetzlar, Germany) for 20 min at 100 °C. The sections were sequentially incubated with an endogenous peroxidase block for 5 min, primary antibody for 30 min, secondary detection polymer for 10 min, diaminobenzidine for 10 min, and hematoxylin for 5 min. The PTC density was evaluated as described previously, with some modifications [11]. Twenty randomly selected fields encompassing the renal cortex and outer medulla were captured using digital imaging (400×). Each image was divided into 270 squares using a grid, and the number of squares with PTCs with CD31-positive staining was counted. Squares with PTCs with CD31-negative staining were not counted. PTC density was expressed as the percentage of CD31-positive squares per total number of squares. If the CD31-positive staining squares were 30 and the total number of squares was 270, PTC density was calculated as 11.1%. Photographs of 20 fields were observed for each rat, and PTC density was calculated in five rats per group.

Macrophages were identified using a rabbit polyclonal anti-F4/80 antibody (1:300; ab100790; Abcam, Cambridge, UK). Photographs of 50 glomeruli were obtained for each rat, and F4/80-positive macrophages were counted in five rats per group.

### 2.5. RNA Extraction and Library Preparation

Total RNA was purified using the Maxwell^®^ RSC RNA extraction instrument according to the manufacturer’s protocol (Promega Corporation, Madison, WI, USA). After fragmentation, first-strand cDNA was synthesized using random hexamer primers, followed by second-strand cDNA synthesis using either dUTP for the directional library or dTTP for the non-directional library [20]. A non-directional library was prepared after end repair, A-tailing, adapter ligation, size selection, amplification, and purification. A directional library was prepared after end repair, A-tailing, adapter ligation, size selection, USER enzyme digestion, amplification, and purification. Library quality and size distribution were assessed using Qubit (Thermo Fisher Scientific, Waltham, MA, USA) and a bioanalyzer (Agilent, Santa Clara, CA, USA). The quantified libraries were pooled and sequenced on an Illumina platform (Novaseq 6000, Illumina, San Diego, CA, USA) at 150 bp according to the effective library concentration and amount of data.

### 2.6. Bioinformatics Analysis

Differential expression analysis of the two conditions/groups (two biological replicates per condition) was performed using the DESeq2 R package (ver. 1.20.0) [21], which provides statistical routines for determining differential expression in digital gene expression data, using a model based on a negative binomial distribution. The resulting *p*-values were adjusted using Benjamini and Hochberg’s approach to control for the false discovery rate (FDR). Following DESeq2, genes with an adjusted *p* < 0.05 were considered differentially expressed. Prior to the differential gene expression analysis, read counts were adjusted for each sequenced library using a scaling factor in the edgeR package. Differential expression was further assessed under the two conditions using the edgeR package (version 3.22.5). The *p*-values were adjusted using the Benjamini–Hochberg method. A corrected *p*-value of 0.05 and an absolute fold change of 2 were set as the thresholds for significantly differential expression. Differentially expressed genes were subjected to functional enrichment analysis using Gene Set Enrichment Analysis to identify coordinated biological pathways associated with the phenotype of interest. Normalized enrichment scores and FDR q-values were computed based on 1000 phenotype permutations, using curated gene sets from the Molecular Signatures Database (MSigDB v7.5). To infer the cellular origin of gene expression changes, cell type enrichment analysis was conducted using the Enrichr platform (https://maayanlab.cloud/Enrichr/ (accessed on 25 January 2024)). Enrichment was assessed against the Human Gene Atlas dataset, which profiles gene expression signatures across various human tissues and cell types. Enrichment scores were calculated using Fisher’s exact test, and combined scores were reported to prioritize the cell types most associated with the input gene list. Only enriched terms with adjusted *p*-values < 0.05 were considered statistically significant.

### 2.7. Statistical Analysis

Data are expressed as the mean ± standard deviation. Group comparisons were performed using analysis of variance (ANOVA), and changes over time in body weight, blood glucose, and UACR during the study period were analyzed using repeated-measures ANOVA. Statistical analysis was performed using SPSS version 11.0 software (SPSS Inc., Chicago, IL, USA), and a *p*-value < 0.05 was considered statistically significant.

## 3. Results

### 3.1. Blood Glucose and Body Weight

**Figure 2:**
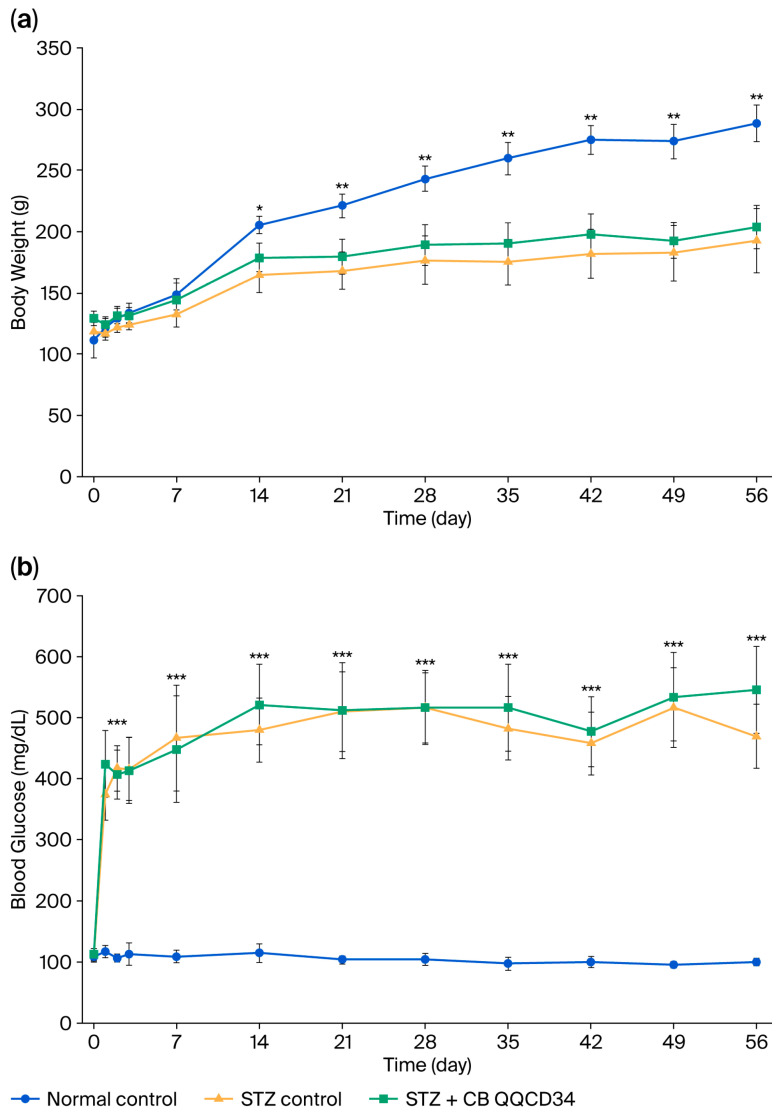
Changes in body weight and blood glucose. (**a**) Body weight. (**b**) Blood glucose. Closed circles, normal control group; closed triangles, STZ control group; closed squares, STZ-plus-cell-therapy group. * *p* < 0.05, ** *p* < 0.01 vs. normal control group; *** *p* < 0.001 vs. normal control group. STZ, streptozotocin.

Although body weight gradually increased in both the normal control and STZ-treated rats, the STZ-treated rats gained less weight than the normal control rats, with significant differences from weeks 3 to 8 post STZ administration (8 weeks: normal control group, 288.4 ± 14.9 g; STZ control group, 192.7 ± 26.2 g; STZ-plus-cell-therapy group, 203.8 ± 17.7 g) (Figure 2a).

Blood glucose levels were significantly elevated within three days of STZ administration. The STZ-treated rats exhibited mean blood glucose levels ranging between 400 and 550 mg/dL during the study period. CD34^+^ cell administration did not affect the blood glucose levels compared with STZ administration (8 weeks: normal control group, 100.0 ± 6.0 mg/dL; STZ control group, 546.0 ± 71.0 mg/dL; STZ-plus-cell-therapy group, 469.0 ± 53.0 mg/dL) (Figure 2b).

### 3.2. Urinary Albumin Excretion and Kidney Function

**Figure 3:**
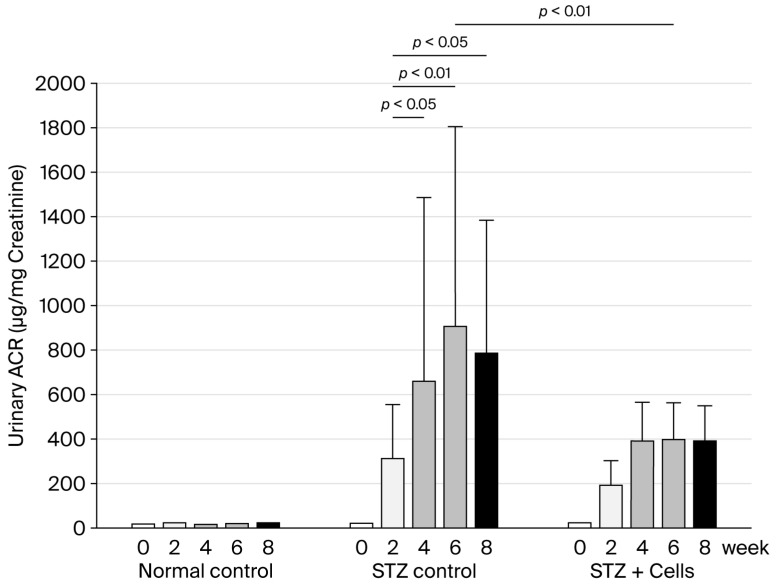
Urinary albumin/creatinine ratio. The UACR progressively and significantly increased in the STZ control group post-STZ administration. The UACR in the STZ-plus-cell-therapy group also increased mildly post-STZ administration. However, it did not differ significantly from the baseline UACR at week 0. Unfilled bar, week 0; light grey bar, week 2; medium grey bar, week 4; dark grey bar, week 6; filled bar, week 8. STZ, streptozotocin; UACR, urinary albumin creatinine ratio.

During the study period, the UACR in the normal control rats was <100 µg/mg creatinine. In the STZ control group, the UACR was significantly higher at week 4 post-STZ administration (631.4 ± 867.6 µg/mg creatinine) than that at week 0 (71.9 ± 71.3 µg/mg creatinine, *p* < 0.05) and increased persistently until the end of the study. Although the UACR was mildly elevated in the STZ-plus-cell-therapy group (306.5 ± 336.8 µg/mg creatinine) at week 4, the difference was not statistically significant when compared with the value at week 0 (27.1 ± 12.4 µg/mg creatinine). The STZ-plus-cell-therapy group had a significantly lower UACR (284.2 ± 303.2 µg/mg creatinine) at week 6 than the STZ control group (991.4 ± 989.1 µg/mg creatinine, *p* < 0.001) (Figure 3).

**Figure 4:**
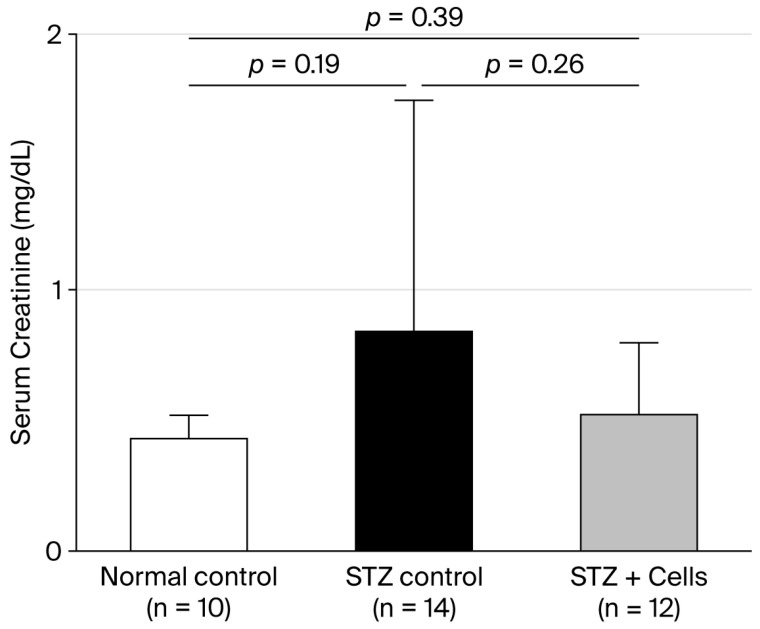
Serum creatinine levels. Unfilled bar, normal control group; filled bar, STZ control group; grey bar, STZ-plus-cell-therapy group. Serum creatinine levels tended to improve in the STZ-plus-cell-therapy group compared with those in the STZ control group. However, the difference was not statistically significant. STZ, streptozotocin.

At week 8, sCr levels were 0.34 ± 006 mg/dL, 0.77 ± 1.00 mg/dL, and 0.43 ± 0.29 mg/dL in the normal control, STZ control, and STZ-plus-cell-therapy groups, respectively. sCr levels tended to be higher in the STZ control group than in the normal control group; however, the difference was not statistically significant. Although sCr levels in the STZ-plus-cell-therapy group tended to decrease compared with those in the STZ control group, the difference was statistically nonsignificant (Figure 4). Creatinine clearance at 8 weeks was 2.12 ± 0.42 mL/minute, 1.80 ± 1.91 mL/minute, and 1.87 ± 2.08 mL/minute in normal control, STZ control, and STZ-plus-cell therapy groups, respectively. Levels of creatinine clearance tended to be lower in the STZ control group and the STZ-plus-cell-therapy group compared with those in the normal group. However, creatinine clearance among the three groups was not statistically different.

### 3.3. Pathological Findings

**Figure 5:**
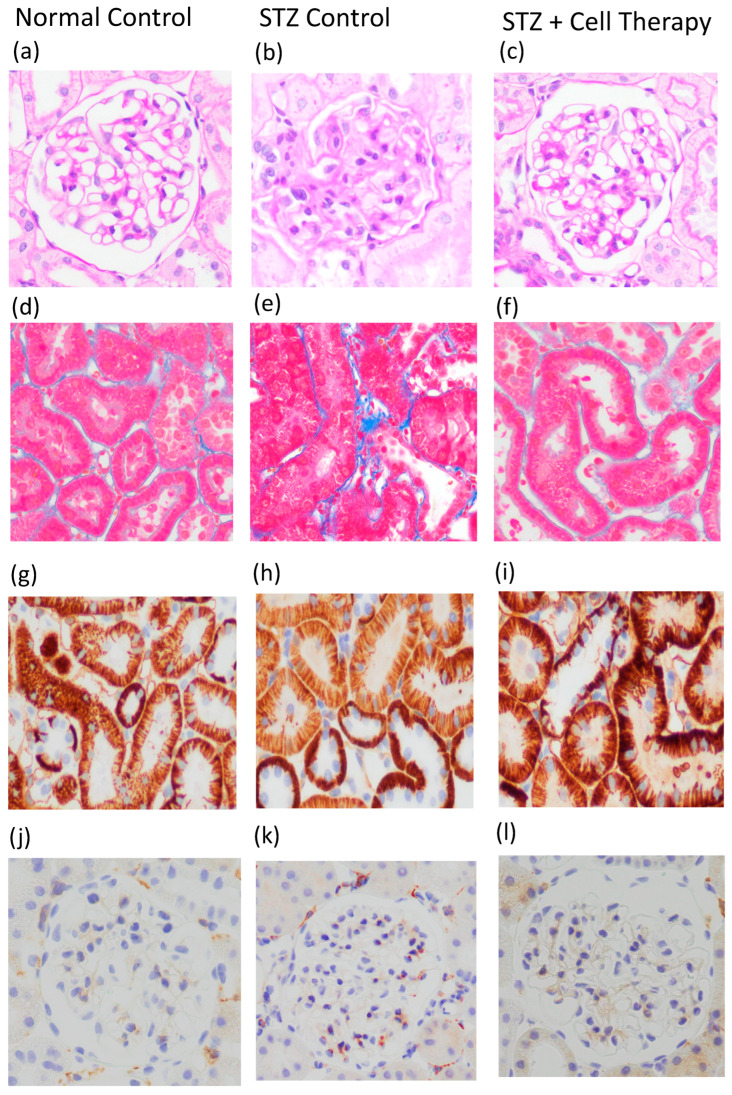

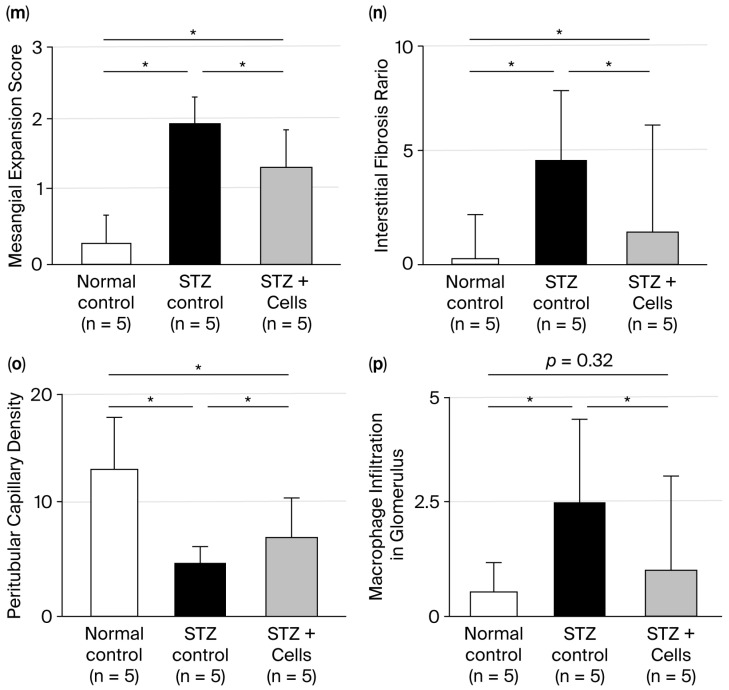
Pathological findings. (**a**–**c**) PAS staining; (**d**–**f**) MT staining; (**g**–**i**) CD31 immunostaining, (**j**–**l**) F4/80 immunostaining. (**a**,**d**,**g**,**j**) normal control; (**b**,**e**,**h**,**k**) STZ control; (**c**,**f**,**i**,**l**) STZ plus cell therapy; (**m**) mesangial matrix expansion score; (**n**) interstitial fibrosis ratio; (**o**) peritubular capillary density; (**p**) macrophage infiltration into glomeruli. Unfilled bar, normal control group; filled bar, STZ control group; grey bar, STZ + cell therapy group. * *p* < 0.0001. Abbreviations are: PAS; periodic acid–Shiff, MT; Masson’s trichrome; STZ, streptozotocin. Original magnification of photographs ×400.

Representative photographs of mesangial matrix expansion, interstitial fibrosis, PTCs, and macrophage infiltration are shown in Figure 5a–l. Cell administration significantly improved all pathological findings, including mesangial matrix expansion, interstitial fibrosis, PTC rarefaction, and glomerular macrophage infiltration, which are representative pathological changes in diabetes. Mesangial expansion was significantly improved in the STZ-plus-cell-therapy group compared with the STZ control group (1.33 ± 0.65 vs. 1.85 ± 0.63, *p* < 0.0001). The interstitial fibrosis ratio (1.53 ± 1.36 vs. 4.38 ± 2.74, *p* < 0.0001), PTC density (6.3 ± 2.3 vs. 3.4 ± 1.5, *p* < 0.0001), and macrophage infiltration (0.87 ± 0.98 vs. 2.45 ± 1.79, *p* < 0.0001) were all significantly improved in the STZ-plus-cell-therapy group compared with the STZ control group (Figure 5m–p).

### 3.4. Transcriptome Analysis

**Figure 6:**
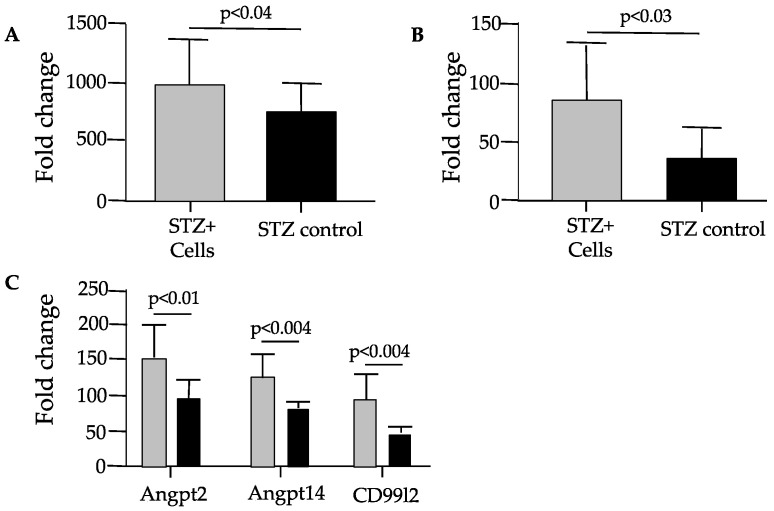
Gene expression in renal tissues. (**A**) M2 macrophage/arginase 2 gene. (**B**) CXCR4 gene. (**C**) Angiogenesis-related genes. Angpt 2, angiopoietin 2; Angpt14, angiopoietin 14; CD99l2, CD 99 molecule-like 2. Gray bar, STZ-plus-cell-therapy group; filled bar, STZ control group. STZ, streptozotocin.

**Figure 7:**
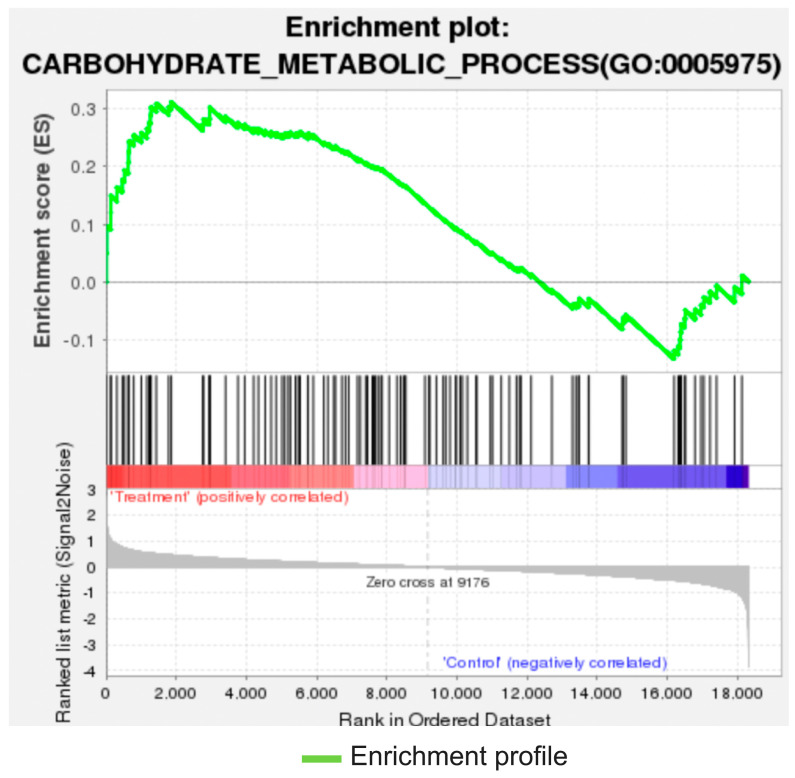
Gene enrichment analysis. Cell therapy significantly enhanced the carbohydrate metabolic pathway activity.

Whole cyclopedic gene expression analysis was performed using renal cortical tissues. Renal tissue transcriptome analysis revealed that the expression levels of the M2 macrophage marker arginase 2 (ARG2), C-X-X motif chemokine receptor 4 (CXCR4), and angiogenesis-related genes (angiopoietin 2, angiopoietin 14, and CD99l2) were significantly upregulated in the STZ-plus-cell-therapy group compared with those in the STZ control group (Figure 6). Gene enrichment analysis revealed that cell therapy significantly enhanced the carbohydrate metabolic pathway activity (Figure 7).

## 4. Discussion

Although STZ administration induced persistent hyperglycemia, repeated administration of human umbilical cord-derived cultured CD34^+^ cells markedly improved urinary albumin excretion and renal pathology, including mesangial matrix expansion, interstitial fibrosis, PTC loss, and glomerular macrophage infiltration, in rats with STZ-induced diabetic nephropathy. The improvement in renal pathology was accompanied by the upregulation of M2 macrophage marker genes, cell homing marker genes, and angiogenesis-related genes. Gene enrichment analysis revealed that the CD34^+^ cell therapy substantially enhanced the carbohydrate metabolic pathway. Thus, cultured human CD34^+^ cell administration improved the phenotype of diabetic nephropathy (urinary albumin excretion and pathological damage), enhanced anti-inflammatory and angiogenic gene expression in kidney tissue, and modified carbohydrate metabolic pathways in a rat model of STZ-induced diabetic nephropathy. Human cultured CD34^+^ cells acted against the injury of diabetic nephropathy in spite of a high blood glucose environment.

In diabetes, hyperglycemia increases the production of reactive oxygen species and advanced glycation end products and decreases nitric oxide synthesis [22]. These changes induce severe endothelial cell injury, a key process in kidney and cardiac injury in patients with diabetes [23]. In addition, both the number and proliferative potential of CD34^+^ EPCs are known to be attenuated in patients with diabetes [23]. These patients also exhibit a reduced capacity for intrinsic repair of injured endothelial cells [23]. Therefore, the extrinsic supply of CD34^+^ cells may be a viable therapeutic approach to treating diabetic endothelial cell injury, as these cells can repair the damaged endothelium and, in this study, markedly protected the PTC network against diabetic injury.

PTC integrity is critical for preserving kidney function. Loss of the PTC network induces tissue ischemia, increases inflammatory cell infiltration and cytokine release, and decreases the mRNA and protein expression of vascular endothelial growth factors and endothelial nitric oxide synthetase activity [24]. This endothelial damage induces microcirculatory impairment and interstitial fibrosis in kidney tissues. Therefore, preserving the microcirculatory environment is critical for retarding or preventing the progression of kidney disease. Given the lack of therapies to restore the PTC network and microcirculation, CD34^+^ cell therapy may be a promising approach for progressive kidney disease.

Whole RNA analysis revealed upregulation of angiogenesis-related (Angpt2, Angptl4, and CD99l2), anti-inflammation-related (ARG2), and cell homing/migration-related genes in the kidney tissues of the STZ-plus-cell-therapy group. The CD34^+^ cell culture technique (QQc) was shown to upregulate gene profiles related to angiogenesis and anti-inflammation in a rat model of myocardial infarction treated with CD34^+^ cell-enriched regeneration-associated mononuclear cells [25]. Administration of these cells preserved cardiac function and fractional shortening at 28 days post myocardial infarction compared with non-cultured mononuclear cell transplantation [25]. Similarly, our results suggest that the administration of cultured CD34^+^ cells may improve kidney tissue by upregulating angiogenesis and anti-inflammation responses, as evidenced by preserved PTC integrity and reduced glomerular macrophage infiltration in the rat model of STZ-induced diabetic nephropathy.

High blood glucose levels did not improve after CD34^+^ cell administration. In the STZ-plus-CD34^+^ cell group, blood glucose levels remained markedly elevated (450–550 mg/dL), indicating a severe and persistent hyperglycemic state consistent with near-complete β-cell destruction. Streptozotocin induces irreversible pancreatic β-cell cytotoxicity, resulting in an absence of endogenous insulin secretion. Although CD34^+^ cell administration led to upregulation of carbohydrate metabolic pathways at the transcriptomic level, these changes most likely reflect metabolic adaptation or enhanced glucose utilization in peripheral tissues rather than restoration of pancreatic endocrine function. Because CD34^+^ therapy did not regenerate β-cells or re-establish insulin production, systemic glucose homeostasis could not be recovered despite molecular activation of metabolic processes. As for regenerative therapy for diabetes, transplantation of human iPSC-derived pancreatic progenitor-derived β-cells rapidly ameliorated diabetes in mice, suggesting strong potential of regenerative medicine for diabetes [26].

In a mouse model of acute ischemia–reperfusion injury, CD34^+^ cell treatment was shown to improve kidney function and pathological damage [27]. Notably, autologous peripheral blood-derived CD34^+^ cell administration was reported to substantially improve severe, acute renal failure in a patient with malignant hypertension who was once dependent on dialysis [28]. In addition to acute kidney injury, CD34^+^ cell therapy may treat chronic kidney injury. As stated earlier, we previously reported that treatment with CD34^+^ cells markedly improved kidney injury in a mouse model of adenine-induced nondiabetic chronic progressive kidney injury [11]. In a clinical trial, autologous CD34^+^ cell therapy for nondiabetic CKD improved the eGFR slope, shifting it from a progressive decline to positive in three out of four patients with progressive CKD [16]. Accordingly, CD34^+^ cell therapy may be a promising and comprehensive therapy for both AKI and CKD. Ischemia and inflammation are key mechanisms driving organ damage in both acute and chronic nondiabetic and diabetic kidney diseases. Therefore, given its robust angiogenic and anti-inflammatory potential, CD34^+^ cell therapy may be a promising regenerative treatment for both nondiabetic and diabetic intractable chronic kidney damage.

We have several limitations in this study. First, we did not perform CD34^+^ cell administration at an earlier phase in STZ-induced diabetic nephropathy. Therefore, the efficacy of early intervention using CD34^+^ cells on the onset of diabetic nephropathy could not be elucidated. However, we wanted to know the effect of human CD34^+^ cells on pre-established overt diabetic nephropathy, in consideration of a future clinical trial for overt diabetic nephropathy in humans. Second, we could not evaluate several pathophysiological aspects of diabetic nephropathy, including oxidative stress, glomerular podocyte injury, the inflammatory cascade of NF-kB, and inflammatory cytokines. We just focused on evaluating whether the strong angiogenic/vasculoprotective and anti-inflammatory potential that CD34^+^ cells possess may alter and improve the progression of diabetic nephropathy. Third, although upregulation of angiogenesis-related genes, M2 macrophage-related genes, and homing-related genes was provided, their protein expression was not evaluated in kidney tissue. Although a lack of enough evaluation about several basic mechanisms of diabetic nephropathy, human CD34^+^ cells could protect against the progression of diabetic nephropathy via angiogenesis and anti-inflammatory related mechanisms.

## 5. Conclusions

To conclude, cultured human CD34^+^ cells markedly improved urinary albumin excretion and attenuated pathological damage in a rat model of STZ-induced diabetic nephropathy, in spite of a hyperglycemic environment, highlighting their angiogenic and anti-inflammatory potential. These findings support the rationale for future clinical trials of CD34^+^ cells in patients with progressive diabetic nephropathy.

## Data Availability

Data are available upon reasonable request.

## Abbreviations

The following abbreviations are used in this manuscript:

ANOVA	Analysis of variance
CKD	Chronic kidney disease
EPC	Endothelial progenitor cell
FDR	False discovery rate
MSC	Mesenchymal stromal cell
STZ	Streptozotocin
UACR	Urinary albumin/creatinine ratio
